# TSPO Modulation Prevents Photoreceptor Degeneration and Produces Neuroprotective Effects in an Animal Model of Retinitis Pigmentosa

**DOI:** 10.3390/cells14221778

**Published:** 2025-11-12

**Authors:** Francesca Corsi, Jacopo Castagnoli, Alessia Galante, Angela Fabiano, Elisa Nuti, Anna Maria Piras, Sabrina Taliani, Ilaria Piano, Claudia Gargini

**Affiliations:** 1 Department of Pharmacy, University of Pisa, 56126 Pisa, Italy; francesca.corsi@farm.unipi.it (F.C.); jacopo.castagnoli@phd.unipi.it (J.C.); alessia.galante@phd.unipi.it (A.G.); angela.fabiano@unipi.it (A.F.); elisa.nuti@unipi.it (E.N.); anna.piras@unipi.it (A.M.P.); sabrina.taliani@unipi.it (S.T.); maria.gargini@unipi.it (C.G.); 2 Center for Instrument Sharing, University of Pisa (CISUP), Lungarno Pacinotti, 43/44, 56126 Pisa, Italy

**Keywords:** neuroinflammation, TSPO, retinitis pigmentosa, PIGA1138

## Abstract

**Highlights:**

**What are the main findings?**

- Topical PIGA1138 preserves retinal function and structure in rd10 mice.
- Treatment modulates TSPO-related pathways, reducing apoptosis and inflammation.

**What are the implications of the main findings?**

- TSPO modulation represents a novel therapeutic avenue for retinal degeneration.
- Topical TSPO ligands show potential for non-invasive neuroprotection in the eye.

**Abstract:**

The translocator protein (TSPO), an evolutionarily conserved protein located on the outer mitochondrial membrane, is typically expressed at low levels in the central nervous system under normal physiological conditions. However, its expression can increase in response to various pathological conditions, such as neurodegenerative diseases and neuroinflammation. Retinitis pigmentosa (RP) refers to a group of inherited degenerative diseases of the retina; the progression of the pathology is linked to a chronic inflammatory state that leads to the progressive loss of photoreceptors and ultimately to blindness. One of the key processes contributing to the gradual loss of photoreceptors is neuroinflammation, a mechanism in which the TSPO plays a newly studied role. In this context, TSPO could be an excellent target. In the current study, rd10 mice of both sexes were treated with a TSPO ligand, PIGA1138, as an ophthalmic suspension (1 mg/mL) from post-natal day (P)18 to P30, P60, and P90. Retinal function was evaluated through electroretinography, while visual acuity was assessed using the Prusky Water Maze task. Additionally, molecular analyses were performed to assess TSPO expression, alongside examinations of retinal morphology. Results showed significant retinal preservation, reduced photoreceptor loss, and improved retinal responses, suggesting preserved visual function. These findings highlight PIGA1138’s potential in mitigating retinal degeneration and preserving function in retinal diseases like RP.

## 1. Introduction

Inflammation is a complex adaptive response of the immune system aimed at eliminating harmful stimuli and restoring physiological homeostasis. While a controlled inflammatory response is generally beneficial, persistent or dysregulated inflammation can become chronic and lead to irreversible tissue and organ damage due to excessive release of pro-inflammatory mediators. In the central nervous system (CNS), an immunologically unique tissue, microglial cells play a crucial role in initiating and modulating inflammation. Under physiological conditions, microglia exhibit a resting (M2) phenotype associated with the release of anti-inflammatory and neurotrophic factors. However, in response to pathogens or tissue injury, they switch to an activated (M1) phenotype, characterized by the production of pro-inflammatory cytokines such as IL-1β, IL-6, IL-12, and TNF-α, as well as chemokines that recruit additional immune cells [1,2].

During chronic neuroinflammatory states of the CNS, prolonged glial cell hyperactivation has been associated with the overexpression of the 18 kDa Translocator Protein (TSPO), a transmembrane protein primarily located on the outer mitochondrial membrane of steroid-synthesizing cells. Physiologically, TSPO takes a crucial role in the transfer of cholesterol from the cytoplasm into mitochondria, a critical step in steroid biosynthesis. Once at the inner mitochondrial membrane, cholesterol is converted by CYP11A1 into pregnenolone, the precursor of anti-inflammatory neurosteroids [3,4]. Beyond steroidogenesis, TSPO interacts with various mitochondrial proteins and is implicated in heme synthesis, mitochondrial energy metabolism, reactive oxygen species (ROS) generation, autophagy, and apoptosis. Although the TSPO contribution to the modulation of the mitochondrial permeability transition pore (mPTP) is debated [5]. Experimental studies have demonstrated that selective TSPO ligands can modulate mPTP opening, preventing or inducing mitochondrial membrane potential loss and subsequent cytochrome c release, thus controlling apoptosis initiation [6,7]. Under healthy conditions, TSPO expression in brain tissue is low but markedly increases at sites of CNS injury or pathology, making it a promising biomarker for assessing reactive gliosis [8]. TSPO overexpression observed in multiple neuroinflammatory models highlights its functional role in microglial activation, proliferation, migration, and phagocytosis, crucial for the innate immune response to CNS injury [9]. Indeed, selective TSPO ligands exhibit immunomodulatory and neuroprotective properties by reducing microglial activation, suppressing pro-inflammatory cytokine expression and secretion, limiting ROS production, and enhancing pregnenolone synthesis [10]. In contrast, TSPO knockdown in microglial cells leads to increased ROS and pro-inflammatory mediator production, while TSPO overexpression reduces NF-κB activation and promotes M2 anti-inflammatory microglial gene expression [11].

In this context, TSPO stands out as a promising therapeutic target to promote neuroprotection [10]. Altered TSPO expression has been observed in a wide range of neurodegenerative and neuroinflammatory diseases, including Parkinson’s disease, Huntington’s disease, amyotrophic lateral sclerosis, Alzheimer’s disease, and several neuropsychiatric disorders [12]. Moreover, TSPO has been implicated in the pathogenesis and progression of several ocular disorders, such as age-related macular degeneration (AMD), diabetic retinopathy (DR), retinal ischemia, and glaucoma, where it appears to exert both protective and detrimental effects depending on the disease context. Growing evidence suggests that endogenous and synthetic ligands targeting TSPO can confer therapeutic benefits in these conditions by enhancing steroidogenesis, preserving cholesterol balance, reducing oxidative stress and inflammation, and modulating microglial activation [9]. In addition, the therapeutic potential of targeting TSPO for the treatment of inflammation-based retinal neurodegeneration was demonstrated in our recent study [13], through the biological evaluation of TSPO ligands from the *N*,*N*-dialkyl-2-arylindol-3-ylglyoxylamide (PIGA) class in an in vitro model.

PIGAs represent a class of synthetic indole-based compounds, developed by some of us as high-affinity and selective ligands for TSPO [14]. Among them, PIGA1138 has demonstrated the ability to effectively modulate TSPO, exerting neuroprotective and anti-inflammatory properties, with an in vivo safety profile [15,16,17,18], thus emerging as a promising therapeutic candidate. Furthermore, in vitro studies on 661 W retinal cells [13] support its potential application in ocular conditions with an inflammatory component, such as cone secondary death in retinitis pigmentosa (RP) [19].

The present study aimed to evaluate the efficacy of this class of TSPO ligands (PIGAs) in promoting neuroprotection in the rd10 mouse model of retinitis pigmentosa (RP). In particular, the study focused on the topical application of an ophthalmic suspension of PIGA1138, taken as representative of the PIGA class, to explore the potential of modulating TSPO activity to elicit neuroprotective effects able to counteract the progression of neurodegeneration. Results from this investigation may pave the way for the development of new therapeutic approaches for the treatment of inherited retinal dystrophies (IRDs).

## 2. Materials and Methods

### 2.1. Animal

The rd10 mouse, affected by a mutation of phosphodiesterase 6b (Pde6brd10/rd10) with a C57Bl/6 J genetic background, was used as a model of retinal neurodegeneration. Degeneration of rod photoreceptors begins around post-natal day 18, followed by degeneration of cone photoreceptors, which is complete at p60 [20]. The mice were kept at a constant room temperature and exposed to a 12 h light/dark cycle, ensuring that the light intensity did not exceed 60 lux. All procedures on these animals were performed in accordance with the ARVO Statement on the Use of Animals in Ophthalmic and Visual Research and Italian and European standards and were approved by the Italian Ministry of Health and the Ethics Committees of the University of Pisa (No. 719/2022-PR—date November 2022) and the principles of the 3Rs.

### 2.2. PIGA1138 Ophthalmic Suspension and Treatment

**Figure 1:**
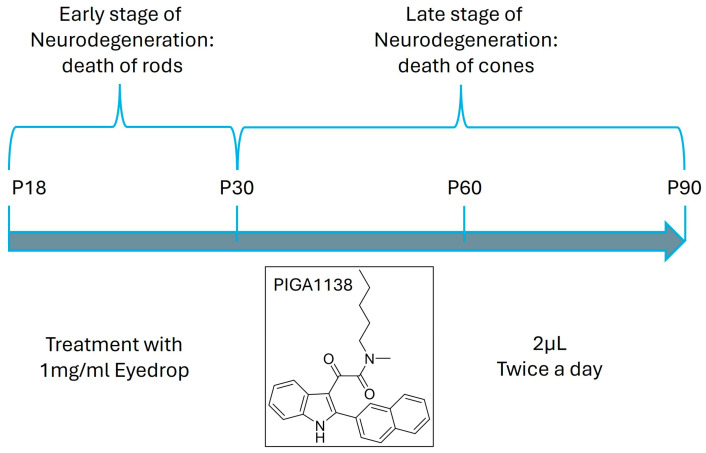
Experimental protocol. rd10 mice were treated with PIGA1138 1 mg/mL eye drops twice daily starting on P18, up to three different time points (P30, P60, P90) that mark the different stages of retinal degeneration in the animal model.

PIGA1138 (Figure 1) was synthesized according to a previously reported procedure [14]. Briefly, 2-(2-naphthyl)indole was first reacted with oxalyl chloride in anhydrous diethyl ether and then treated with N-methyl-N-pentylamine, in the presence of triethylamine in anhydrous toluene. Working out of the reaction mixture furnished the desired PIGA1138, whose chemistry and purity were verified by spectroscopic and analytical data 16.

The ophthalmic vehicle consisted in 5 mg/mL Kolliphor RH40 (Merck, Darmstadt, Germany), dissolved in sterile saline solution and filtered through 0.45 µm cellulose membrane filters. Kolliphor^®^ RH 40, a non-ionic solubilizer widely used in ophthalmic products, was incorporated at 0.5% *w*/*w*, in line with concentrations reported for approved eyedrop formulations, in the FDA Inactive Ingredients Database (last accessed on 9 December 2025) (https://www.fda.gov/). PIGA1138 was sterilized via UV irradiation for one hour before being dispersed in Kolliphor solution, at a final concentration of 1.0 mg/mL. The preparation was performed under a flow laminar sterile lab hood (Thermo Scientific MSC-Advantage, Milan, Italy). The suspension was stirred at 50 °C for 4 h until a homogeneous suspension was obtained. Particle diameter distribution resulted monomodal distribution, with an average diameter of 1.352 ± 0.044 μm and PDI of 0.677 ± 0.031, as determined by Dynamic Light Scattering (DLS-Zeta Sizer Nanon Series instrument, Malvern Panalytical Ltd., Malvern, UK) at 25°. Z-average is well below the particle size limits specified by both the European Pharmacopeia (Ph. Eur.) [21] and the United States Pharmacopeia (USP) [22], thereby minimizing the risk of ocular irritation and excessive tearing. Isotonicity was also verified (297 mOsm/Kg; Osmomat 3000, Gonotec, Berlin, Germany) and ensures that the vehicle itself does not contribute to ocular discomfort.

The concentration selected for topical administration was based on dosage previously reported in Tremolanti et al., 2022 [23]. In the absence of specific ocular pharmacokinetic data, this systemic regimen was used as a reference to guide dose selection. Considering the low retinal bioavailability generally associated with topical administration, reported to be below 0.1% of the instilled dose [24], it was not possible to determine whether sufficient drug levels would reach the posterior segment. To address this uncertainty, a 10- to 20-fold increase over the estimated systemic-equivalent dose was applied. This level was chosen as the maximum concentration compatible with formulation stability, as higher concentration led to visible precipitation during development.

Treatment began at P18, simultaneously with the onset of rod degeneration, and continued at three different time points: P30, P60, and P90.

The animals, totaling 62 and of both sexes, were previously divided into two groups: a control group, which received the formulation without PIGA1138, and a treated group, which received the formulation containing PIGA1138 at a concentration of 1 mg/mL. Treatment began at P18, coinciding with the onset of rod degeneration, and continued at three different time points: P30 (ctrl n = 11, PIGA1138 n = 10), P60 (ctrl n = 10, PIGA1138 n = 10), and P90 (ctrl n = 10, PIGA1138 n = 11).

The animals were treated twice daily with 2 µL of ophthalmic solution containing PIGA1138 at a concentration of 1 mg/mL.

### 2.3. Behavioral Test: Prusky Water Maze

The Prusky Water Maze test was used with the same set of animals at different ages (30, 60, and 90 days) to evaluate visual acuity under photopic conditions [25]. The visual stimuli were computer-generated black-and-white square-wave gratings with spatial frequencies ranging from 0.087 to 0.550 cycles/degree and a fixed luminance of 39.95 cd/m^2^. These stimuli were created with MATLAB 2022 and PsychToolbox-3 and shown on gamma-linearized monitors.

The mice underwent initial training at 20 days old, the spatial frequency was progressively changed to determine the threshold of each animal in locating the platform. To increase the spatial frequency of the stimulus, one cycle was added to the screen to see if the animal made the correct choice during the test. This procedure was repeated for the low spatial frequencies until an error occurred, minimizing the amount of time away from the threshold. After an error, extra trials were conducted until either four consecutively accurate responses were generated or seven correct choices were provided in a block of 10 trials.

As a measure of visual acuity, the highest spatial frequency at which the platform was detected correctly, 70% of the time, was noted.

### 2.4. Electroretinogram (ERG)

A 0.1 mL/10 g body weight intraperitoneal injection of urethane (20% physiological saline solution) was used to anesthetize the dark-adapted mice; then 2 µL of Sigma’s 0.5% atropine was administered. A coating of methylcellulose (Lacrinorm, Farmigea, Pisa—Italy) was placed on the cornea to keep it moistened throughout the analysis. Every mouse was set up on a 37 °C-heated Diagnosys Celeris rodent-ERG device (Diagnosys LLC, Lowell, MA, USA). The ground electrode was placed in the back of the tail, while the reference electrode was placed subcutaneously at the head. Two recording electrodes were then positioned at the corneal surface of each eye. Light stimulation was performed using the Diagnosys Celeris rodent-ERG device (Diagnosys LLC).

The animals were subjected to nine different light intensities (0.004–377 cd∗s/m^2^) first in dark adaptation conditions (scotopic ERG) and then, after 15 min of adaptation to a constant background of 30 cd/m^2^, the nine light intensities were superimposed. The background is necessary to simulate photopic conditions and saturate the response of the rods, so that only the electrical activity of the cones (photopic ERG) is recorded.

The data were analyzed using Espion V6 software (Diagnosys LLC)). For ERG analysis, both eyes of each mouse were recorded under identical conditions. The b-wave amplitudes obtained from the right and left eyes were averaged to generate a single representative value per animal, which was then used for statistical comparisons.

### 2.5. Immunohistochemistry

Whole eyes were collected from mice treated with PIGA1138 and control mice at 30, 60, and 90 days of age. A total of 18 mice (3 for each group) were used for immunohistochemistry.

The immunostaining on the whole mount was performed as reported by [26]. Retinal whole mounts were first labeled for cell death using DeadEnd™ Fluorometric TUNEL System, Alexa Fluor™ 488 dye (Promega G3250, Madison, WI, USA). After incubation with the blocking solution, the primary antibody Cone arrestin (Millipore, Burlington, MA, USA, Rabbit 1:200) was added to the retinas and incubated at 4 °C for 3 days in a 1% BSA and 0.1% Triton solution. The retinas were then treated with the secondary antibody (Bio-Rad, Hercules, CA, USA, anti-Rabbit 568) for 2 days. Finally, the retinas were opened in whole mount, placed on microscope slides with the photoreceptor side up, and coated with Vectashield. Nikon mod. NiE fluorescent microscope with Nikon model. A DS-Ri2 digital camera was used to capture the images. TUNEL+ cells and Cone cells counting was performed by acquiring images at 20× on the focal plane of the outer segments of the cones. For each sample, 6 different regions of the retina, spaced along the dorso-ventral and naso-temporal meridians, were acquired. Images and counting were performed by the NIS-Elements Ar 6.2 software. The percentage of apoptotic cells was calculated as the ratio between TUNEL-positive cells and cone arrestin-positive cells within the same retinal area. Representative images showing cone morphology were acquired using a Nikon confocal microscope equipped with a 40× water-immersion objective and a 1.5× digital zoom to improve visualization of cone structure.

Vertical retinal sections were prepared as previously described [26]. Sections were incubated with DAPI solution in PBS (1:5000, Sigma-Aldrich, Merck Group, Burlington, MA, USA) to visualize nuclei and subsequently mounted with Vectashield mounting medium (Vector Laboratories, Inc., Newark, NJ, USA).

Images were acquired using a Nikon confocal microscope. For the quantification of nuclear rows in the outer nuclear layer (ONL), a 20× air objective was used, and three retinal sections were analyzed for each eye. Representative images shown in the figures were captured using a 40× water-immersion objective with a 1.5× digital zoom to enhance visualization of retinal morphology.

### 2.6. Western Blot

Protein lysates were obtained using a modified RIPA lysis buffer (50 mM Tris-HCl, pH 7.4, 150 mM NaCl, 1% NP-40, 0.25% p/v Sodium deoxycholate, 1 mM EDTA, 2 mM PMSF, and 2 mM Na_3_VO_4_—all from Sigma-Aldrich), adding of 1 µL to 100 µL of Protease Inhibitor Cocktails (Sigma-Aldrich). Following the manufacturer’s instructions, samples were quantified using the Bradford assay (Bio-Rad). It has already been explained how to run protein samples through an electrophoretic, incubate antibodies, and analyze the results 14. Briefly, the Laemmli 2X (Bio-Rad) solution was combined with 25 μg of each cell protein extract, and a precast stain-free gel (Bio-Rad) was used to support the electrophoresis SDS-PAGE.

After activation, the separated proteins were transferred to PVDF membranes (Bio-Rad). The membranes were incubated with the antibody. All the proteins of interest were normalized to the total protein content [27]. The densitometry analysis was undertaken using Bio-Rad ImageLab software 6.0 (Bio-Rad).

Antibodies and dilutions used are listed below: Anti-Bcl2 (Cell Signaling, Danvers, MA, USA, Rabbit 1:1000), Anti-Beclin1 (Cell Signaling, Rabbit 1:1000), TSPO (Invitrogen, Waltham, MA, USA, Rabbit 1:1000) and Anti-Rabbit (Merck Millipore 1:5000).

### 2.7. Statistical Analysis

Statistical analyses of ERG parameters were performed using Origin Lab 8.0 with a one-way ANOVA, followed by *t*-tests adjusted using the Bonferroni method (MicroCal, Northampton, MA, USA). For all other datasets, analyses were conducted with GraphPad Prism 8, applying either parametric or non-parametric tests according to the outcomes of normality assessments. Details of the statistical procedures applied are provided in the figure legends. A threshold of *p* < 0.05 was adopted to indicate statistical significance. All animals used in this study were included in the statistical analysis.

## 3. Results

### 3.1. Assessed Visual Acuity in Animals at Different Stages of Neurodegenerative Disease Progression by Prusky Water Maze Behavioral Test

**Figure 2:**
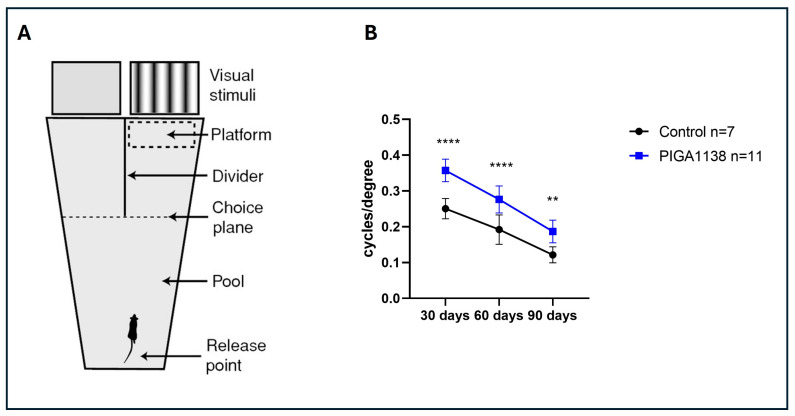
Visual acuity was assessed by the Prusky water maze test. (**A**) Visual water box used for determining the visual acuity in the mice (modified from [25]). (**B**) Visual acuity is expressed as cycles/degree in mice treated with PIGA1138 eye drops and in the control mice at different time points (P30, P60, P90). The mice were divided into 2 groups: control (n = 7) and PIGA1138 (n = 11); the same animal performed the test at different time points. Data is presented as mean ± SD. Two-way ANOVA followed by Tukey’s multiple comparison test; ** *p* = 0.0051 (PIGA1138 P90 vs. ctrl P90) **** *p* = 0.0001 (PIGA1138 P60 vs. ctrl P60); **** *p* = 0.0001 (PIGA1138 P30 vs. ctrl P30). F (2, 46) = 81.59.

The Prusky Water Maze (PWM) is a behavioral test designed to assess spatial visual acuity in rodents, based on their ability to discriminate visual patterns to locate a hidden escape platform. It is widely used to evaluate functional vision in models of retinal degeneration. Here, the results of the PWM test (Figure 2) indicate that PIGA1138 treatment effectively preserves visual acuity in rd10 mice, mitigating the progressive decline associated with retinal degeneration. At 30 days, the treated group showed a significantly higher spatial visual acuity compared to the control group, suggesting an early protective effect of PIGA1138 on retinal function. This effect remained significant at 60 days, indicating sustained efficacy over time. Although the difference between the two groups was still present at 90 days, the gap was decreasing, likely due to the natural progression of retinal degeneration.

### 3.2. Assessed Retinal Function Preservation

**Figure 3:**
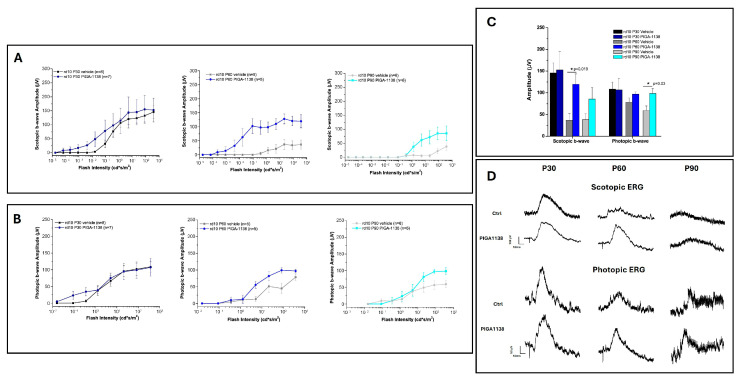
Assessment of retinal function by ERG recording. (**A**) b-wave amplitudes as a function of the light intensity of the ERG flashes scotopic relative to the groups treated with PIGA1138 (blue curves) and respective controls (black curves) at P30, P60 and P90. (**B**) b-wave amplitudes as a function of light intensity of photopic ERG flashes relative to groups treated with PIGA1138 (blue curves) and respective controls (black curves) at P30, P60 and P90. (**C**) Scotopic and photopic b-wave amplitudes at the highest light intensity (377 cd∗s/m^2^) relative to the groups treated with PIGA1138 (blue bars) and the respective controls (black and grey bars) at P30, P60, P90. (**D**) Representative trace of ERG recording at the highest light intensity (377 cd∗s/m^2^). The rd10 specimens were divided into 6 different groups: PIGA1138 P30 (n = 7), control P30 (n = 8), PIGA1138 P60 (n = 5), control P60 (n = 5), PIGA1138 P90 (n = 6), control P90 (n = 6). Statistical comparisons we performed with one-way ANOVA analysis followed by Bonferroni’s correlation *t*-test, mean ± SEM. * *p* ≤ 0.05 (Scotopic b-wave PIGA1138 P60 vs. Ctrl P60; photopic b-wave PIGA1138 P90 vs. Ctrl P90). F (1,10).

Then, the response of photoreceptors to different light stimuli was investigated by ERG. Figure 3 presents scotopic (rod-mediated) and photopic (cone-mediated) ERG responses in rd10 mice treated with either PIGA-1138 or vehicle control at different time points (P30, P60, and P90). In both recordings, the parameter that was measured to assess the efficacy of the treatment was b-wave, because at the chosen time points, in the rd10 animal model, a-wave cannot be evaluated [20].

At P30, the scotopic b-wave amplitude is the same in both PIGA1138-treated (red) and control group (black) mice, at all flash intensities. This trend suggests that PIGA1138 is not able to preserve the rods from early degeneration due to a genetic mutation. At P60, the protective effect of PIGA1138 becomes evident, with treated mice showing higher scotopic amplitudes than controls, although the difference appears to stabilize at higher light intensities. By P90, the scotopic response in both groups is reduced, indicating the progressive loss of rod function (Figure 3A). However, PIGA1138-treated mice still exhibit a slightly better response than controls, suggesting residual rod activity. The photopic b-wave amplitude follows a similar trend. At P30, PIGA1138-treated mice exhibit overlapping cone responses to controls; at this stage of the disease, the cones have not yet begun to degenerate, so the protective activity of PIGA1138 is reasonably not detectable. At P60, the difference between the two groups remains, but the gap is less pronounced compared to the scotopic response, suggesting a relatively slower decline in cone function. At P90, both groups show reduced photopic responses, but the PIGA1138-treated mice maintain higher amplitudes, suggesting prolonged cone survival.

### 3.3. Anti-Apoptotic Activity and Retinal Morphology Preservation After PIGA1138 Treatment

**Figure 4:**
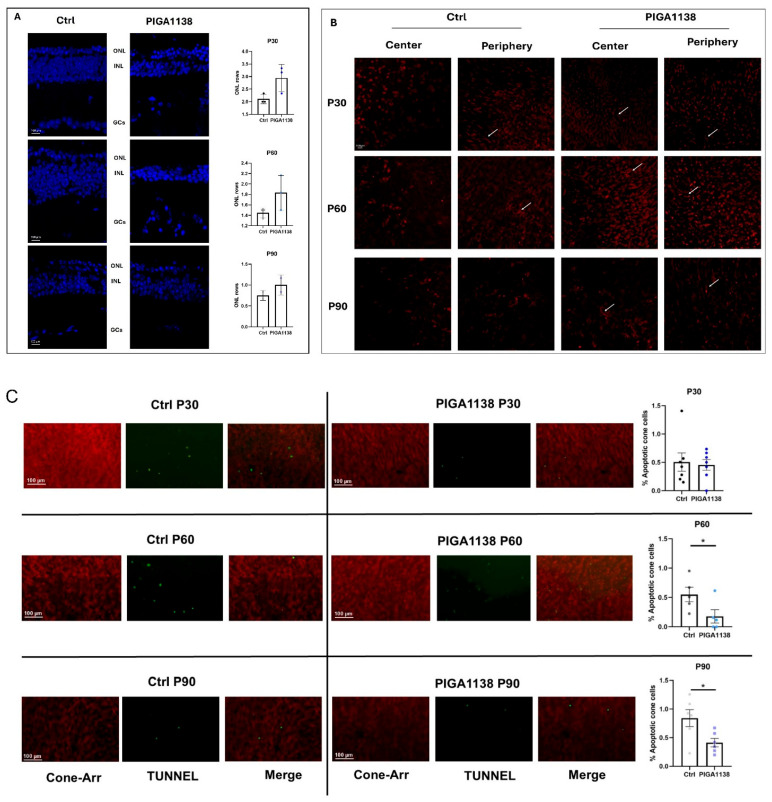
Cone Cell Survival Over Time. (**A**) Representative DAPI-stained retinal sections from control (Ctrl) and PIGA1138-treated mice at P30, P60, and P90 are shown (blue fluorescence). Outer nuclear layer (ONL), inner nuclear layer (INL), and ganglion cell layer (GCs). Quantification of ONL thickness, expressed as the number of nuclear rows, is reported in the bar graphs. Data are presented as mean ± SEM (n = 3 per group). (**B**) Representative retinal whole mounts from control (Ctrl) and PIGA1138-treated rd10 mice at P30, P60, and P90 were immunostained for cone arrestin (red) to visualize cone photoreceptors. Images were captured from both central and peripheral retinal regions, as indicated. Arrows point to representative cone outer segments. (**C**) High-magnification fluorescence images of retinal whole mount stained for cone arrestin (cones) and TUNEL (apoptotic cells) across all experimental groups; and quantification of apoptotic cone cells, expressed as the percentage of TUNEL-positive cells relative to cone arrestin-positive cells in each group. Data are presented as mean ± SEM (n = 3 per group). Comparisons between groups were performed using an unpaired Student’s *t*-test (two-tailed). * *p*  ≤  0.5 (PIGA1138 P60 vs. Ctrl P60; PIGA1138 P90 vs. Ctrl P90). Scale bar: 100 µm.

Figure 4 illustrates the neuroprotective effect elicited by PIGA1138 treatment. In particular, Figure 4A shows a trend toward the preservation of photoreceptor cell number, quantified as rows of nuclei within the outer nuclear layer. Although the increase in cell number does not reach full recovery, the most striking effect emerges in the morphology of the surviving cone photoreceptors. As shown in Figure 4B, treated retinas display not only a greater number of cones, but these cells also retain a more defined and physiologically relevant structure. The white arrows highlight the preservation of cone outer segments, a key structural feature required for phototransduction and thus for maintaining visual function. This morphological preservation strongly suggests that PIGA1138 is not merely slowing cell loss but actively maintaining photoreceptor integrity. This observation is further supported by Figure 4C, which shows photoreceptors labeled with cone arrestin (red) and apoptotic cells detected by TUNEL staining (green) in retinal whole mount. Each panel represents a time point analyzed (P30, P60, P90) in both the control and treatment groups. The relative bar graph shows the quantification of TUNEL-positive cells relative to the total number of cones present, evaluated in terms of fluorescence. At P30, it can be seen that the ratio obtained is completely comparable between the control group and the group of animals treated with PIGA1138, indicating that at this stage, in both experimental groups, the peak of rod cell death has been exceeded, and the process of cone degeneration has not been exacerbated. As the degenerative process progresses, the effects of PIGA1138 treatment become more evident, confirming the functional data; in fact, at P60, the ratio between TUNEL+ cells and cones is significantly reduced (* *p* < 0.05) in treated animals, indicating a reduced number of apoptotic cells in favor of a greater number of cones. The same trend can also be seen at P90, indicating the ability of PIGA1138 treatment to preserve cone survival for long periods of time.

Together, these results indicate that PIGA1138 confers functional and structural protection to cone photoreceptors in the rd10 model, thereby supporting the preservation of visual function in rd10 mice.

**Figure 5:**
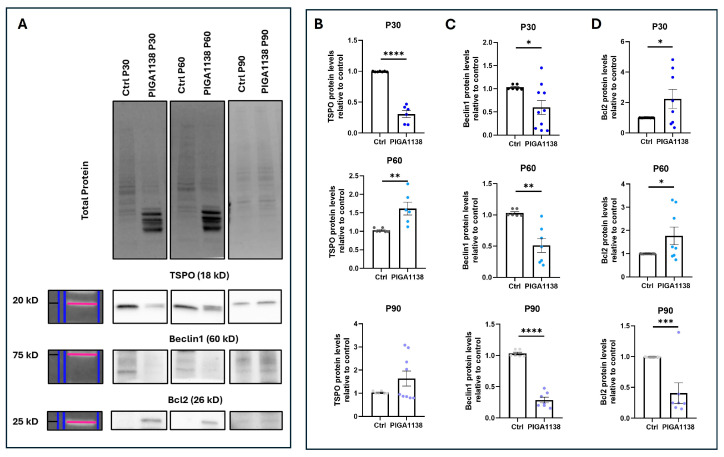
Protein expression analysis in retinal tissue samples. (**A**) Representative Western blot images. (**B**) Bar graphs showing the quantification of TSPO levels assessed by optical densitometry. (**C**) Bar graphs showing the quantification of Beclin-1 protein levels assessed by optical densitometry. (**D**) Bar graphs showing the quantification of Bcl-2 protein levels assessed by optical densitometry. The treated groups receiving PIGA1138 at a concentration of 1 mg/mL are represented by blue and violet bars, while the control groups are shown in black and gray bars at P30, P60, and P90. n = 10 for each group. Data are represented as mean ± SEM; comparisons between groups were performed using an unpaired Student’s *t*-test (two-tailed). * *p*  ≤  0.5 (Beclin1 (*p* = 0.046; DFn = 9) and Bcl2 (*p* = 0.0405; DFn = 7) PIGA1138 P30 vs. Ctrl p30; Bcl2 (*p* = 0.0357; DFn = 7) PIGA1138 P60 vs. Ctrl P60); ** *p*  ≤  0.05 (TSPO (*p* = 0.0075; DFn = 5) and Beclin1 (*p* = 0.0015; DFn = 6) PIGA1138 P60 vs. Ctrl P60); *** *p*  ≤  0.01 (Bcl2 (*p* = 0.0007; DFn = 6) PIGA1138 P90 vs. Ctrl P90); **** *p*  ≤  0.001 (TSPO (*p* = 0.001; DFn = 5) PIGA1138 P30 vs. Ctrl P30; Beclin1 (*p* = 0.0001; DFn = 6) PIGA1138 vs. Ctrl P90).

Following the completion of ERG recordings, retinas from the same animals were harvested and used for subsequent analyses. Using the semi-quantitative analytical technique of Western blotting, we identified, analyzed, and quantified the expression levels of three proteins of interest in lysed retinal samples: TSPO, the primary target of this study as a biomarker of neuroinflammation; the autophagy-related protein Beclin-1; and the anti-apoptotic protein Bcl-2. The latter two proteins are actively involved in the signaling pathways associated with programmed cell death in retinal photoreceptors, particularly in response to homeostatic imbalances such as oxidative stress, inflammation, and mitochondrial damage. For this analysis, retinal samples were collected from both PIGA1138-treated rd10 mice and their respective control groups at the three temporal points (P30, P60, and P90). Figure 5A reports representative Western blot images. The results presented in Figure 5B–D showed graph bars depicting protein quantification at the three time points: blue and violet shades represent protein levels in PIGA11138-treated retinal samples, while black and shades of gray indicate levels in the respective control groups. A significant increase in TSPO levels is observed at P60 and P90 in the PIGA1138-treated groups compared to controls (Figure 5B). Figure 5C showed, at all three time points, a significant downregulation of Beclin-1 expression in the PIGA1138-treated group compared to controls. Figure 5D displays a statistically significant upregulation of Bcl-2 at P30 and P60 in the PIGA1138-treated group compared to the corresponding controls.

## 4. Discussion

Neurodegenerative retinal diseases, particularly retinitis pigmentosa (RP), represent a complex and multidisciplinary research challenge at the intersection of genetics, cell biology, pharmacology, and neuroscience [19,28]. Despite their heterogeneous etiologies and clinical manifestations, these disorders share a unifying pathological hallmark: chronic neuroinflammation, largely driven by sustained microglial overactivation [29]. This persistent inflammatory state plays a pivotal role in the progressive degeneration of the highly specialized neurons within the sensory retina. Accordingly, neuroinflammation has emerged as a promising therapeutic target in the quest to develop novel preventative and disease-modifying strategies for inherited retinal dystrophies [30].

Building on the growing interest in the 18 kDa Translocator Protein (TSPO) as a pharmacological target in retinal neurodegeneration, this study investigated the therapeutic potential of a class of selective TSPO ligands, the *N*,*N*-dialkyl-2-arylindol-3-ylglyoxylamides (PIGAs), administered as eye drops. We focused specifically on PIGA1138, as a promising representative compound belonging to PIGA class, and evaluated its neuroprotective efficacy in the well-characterized rd10 mouse model of RP. A streamlined formulation was designed to maximize ocular tolerability by including only essential excipients. Indeed, no irritation or animal discomfort was recorded, whereas effective results were observed. Efforts on adjusting dose regimes and formulation composition to deepen PIGA1138 biopharmaceutical aspects will be provided in future works. The role of TSPO in cholesterol transport within the mitochondrial membrane, steroidogenesis, and regulation of mitochondrial function have recently emerged as a key element in cellular responses to stress and injury. The ability to pharmacologically modulate TSPO activity arises from the capacity of its ligands to stimulate neurosteroidogenesis (by facilitating cholesterol transport and its conversion into pregnenolone via cytochrome P450) [31,32,33], enhance mitochondrial respiration and ATP production, preserve mitochondrial membrane potential by reducing oxidative stress [9], and downregulate pro-inflammatory mediators, thus promoting a shift in microglia from an activated to a quiescent state [11]. Given its role in modulating mitochondrial homeostasis and glial activation, the main objective of this study was to determine whether pharmacological modulation of TSPO could attenuate retinal degeneration by acting on two key pathological mechanisms: chronic inflammation and oxidative stress, both of which are responsible for photoreceptor death and disease progression. The experimental findings consistently support the therapeutic relevance of PIGA1138. Treated animals displayed significant improvements in both functional and molecular readouts compared to untreated controls.

Behavioral testing using the Prusky water maze demonstrated that photopic visual acuity was significantly preserved in PIGA1138-treated mice across all three evaluated time points. At P30, an early stage of degeneration, treated animals exhibited near-physiological levels of visual acuity, indicating substantial protection of the initial retinal structures affected. Although some functional decline was noted at P60, mice still outperformed controls, whose visual performance was already severely compromised. By P90—when degeneration is typically near complete in rd10 mice—treated animals retained a residual visual response, suggesting that PIGA1138 significantly delayed disease progression. Electroretinogram (ERG) recordings corroborated these behavioral results. Analysis of the b-wave revealed enhanced retinal responses in treated mice, particularly in the earlier stages of degeneration. At P30, higher scotopic and photopic b-wave amplitudes were observed in treated animals in response to the highest light intensity (377 cd∗s/m^2^), indicating better bipolar cell function and, by extension, healthier photoreceptor populations. At P60, although b-wave amplitudes decreased in both groups, they remained significantly higher in the treated group. At P90, while responses were attenuated overall, a residual b-wave persisted in PIGA1138-treated mice, underscoring the compound’s ability to prolong photoreceptor viability beyond the typical degenerative threshold in this model. At the molecular level, Western blot analyses revealed significant differences in the expression of key markers of oxidative stress and microglial activation between treated and untreated groups. Notably, TSPO levels were significantly elevated in the treated animals at P60 and P90—later stages of degeneration—highlighting its relevance as both a therapeutic target and a potential biomarker for reactive gliosis in retinal neurodegenerative conditions. Moreover, treated animals showed a marked increase in the expression of Bcl-2, a well-established anti-apoptotic protein. Upon dissociation from its binding partner Beclin-1, Bcl-2 inhibits photoreceptor apoptosis triggered by inflammatory stress and simultaneously promotes autophagy. Interestingly, across all three time points, Beclin-1 expression tended to decrease significantly in treated animals—likely due to intracellular degradation following its release from the Bcl-2/Beclin-1 complex. This reduction, when coupled with Bcl-2 upregulation, may reflect a shift toward a protective, physiologically regulated autophagic process that aids in retinal repair and survival.

Overall, topic PIGA1138 treatment resulted in the downregulation of pro-inflammatory and pro-apoptotic markers, consistent with its modulatory effects on neuroinflammation. These protective effects were most pronounced at P30 and P60, aligning with the active phase of degeneration. By P90, the differences between groups diminished, likely reflecting the advanced state of retinal damage and the extensive loss of target cells at this late stage.

Taken together, these data underscore the importance of early intervention. The greatest benefit of eye drops formulation of PIGA1138—both functional and molecular-were observed when treatment was initiated at the early stages of retinal degeneration. The compound effectively delayed disease progression, preserved visual function, and reduced molecular hallmarks of neuroinflammation and oxidative stress. Although its efficacy appeared to taper in the later stages, the retention of measurable visual responses at P90 remains an encouraging outcome, especially given the aggressive nature of the rd10 model. These results are in strong agreement with the current literature, which highlights TSPO as an emerging target in neurodegenerative disease due to its involvement in neurosteroid synthesis, ROS regulation, apoptosis, and innate immune responses [3,34,35]. Notably, the long residence time at TSPO of PIGA1138 and its validated ability to stimulate steroidogenesis in vitro further support its potential as a highly effective therapeutic tool [13,36]. While the therapeutic promise of PIGA1138 is evident, further studies are needed to evaluate its long-term efficacy and safety, as well as its potential to synergize with other therapeutic strategies—such as gene therapy, classical neuroprotection, cell transplantation, and retinal prosthetics. Although the observed functional, morphological and molecular improvements strongly suggest retinal engagement of TSPO, dedicated pharmacokinetic and biodistribution studies are required to substantiate this hypothesis. Future studies will include quantitative analyses of ocular distribution using LC–MS/MS, as well as imaging-based assessments of TSPO target engagement. This approach is supported by recent comprehensive reviews highlighting the key role of TSPO in retinal physiology and in various ocular pathologies, where its modulation influences neuroinflammation and photoreceptor survival [37]. Nevertheless, the findings presented here offer a compelling foundation for advancing TSPO-targeting compounds as viable candidates in the treatment of inherited retinal degenerations.

## 5. Conclusions

In conclusion, this work contributes to the growing body of preclinical research on Retinitis pigmentosa by providing new experimental evidence supporting the use of TSPO ligands as a potential therapeutic strategy. Although the path toward clinical application remains long and complex, the approach based on PIGA1138 offers a promising perspective for the treatment of a currently incurable condition. These findings open a hopeful avenue for patients affected by inherited retinal dystrophies, laying the groundwork for future translational studies and the development of innovative, targeted therapies.

## Data Availability

Data will be made available on request.
